# Differences in Transmission between SARS-CoV-2 Alpha (B.1.1.7) and Delta (B.1.617.2) Variants

**DOI:** 10.1128/spectrum.00008-22

**Published:** 2022-04-12

**Authors:** Camino Trobajo-Sanmartín, Iván Martínez-Baz, Ana Miqueleiz, Miguel Fernández-Huerta, Cristina Burgui, Itziar Casado, Fernando Baigorría, Ana Navascués, Jesús Castilla, Carmen Ezpeleta

**Affiliations:** a Instituto de Salud Pública de Navarra, Pamplona, Spain; b CIBER Epidemiología y Salud Pública (CIBERESP), Madrid, Spain; c Navarra Institute for Health Research (IdiSNA), Pamplona, Spain; d Clinical Microbiology Department, Hospital Universitario de Navarra, Pamplona, Spain; University of Mississippi Medical Center

**Keywords:** COVID-19, SARS-CoV-2, Alpha variant, Delta variant, COVID-19 vaccine, transmission, infectivity, susceptibility, close contact

## Abstract

The present study aimed to compare the susceptibility and infectivity between the Alpha and Delta variants of SARS-CoV-2 and to investigate characteristics of the index case and the contact that may affect transmission. The risk of SARS-CoV-2 infection was compared between close contacts of COVID-19 cases with Alpha and Delta variants during June 2021 to August 2021. In index cases, Spike gene target failure (TaqPath) was used as a proxy of Alpha variant and the L452R mutation (TaqMan) for Delta variant. Cox regression models were used to estimate adjusted relative risks (RR). We compared close contacts of index cases with Alpha (*n* = 2139) and Delta variants (*n* = 5439). Delta variant was more transmissible overall (relative risk [RR] 1.32, 95% CI = 1.13 to 1.53), and in non-household contacts (RR 1.71, 95% CI = 1.35 to 2.16), but not in household contacts (RR 1.10, 95% CI = 0.91 to 1.34; *P_interaction_* < 0.001). Delta variant excess transmission was observed when the index cases were 12 to 39 years old (RR 1.51, 95% CI = 1.27 to 1.79) and the close contacts were 18 to 39 years old (RR 1.62, 95% CI = 1.29 to 2.03), but not among those younger or older than such ages. Differences in transmissibility between variants disappeared with vaccination of the index case (RR 0.68, 95% CI = 0.46 to 1.02), but not with vaccination of the close contact. This report shows that the Delta variant is more transmissible than Alpha variant mainly among young adults. Vaccination of the index cases reduced the excess transmission, which reinforces the recommendation of vaccination to reduce transmission of the Delta variant.

**IMPORTANCE** The higher transmissibility of the Delta variant of SARS-CoV-2 in comparison with the Alpha variant has been reported. We compared the transmission of the Alpha and Delta variants by characteristics and COVID-19 vaccination status of index cases and their close contacts. Interestingly, the Delta variant showed increased transmissibility when the index case was an adolescent or young adult and when the close contact was a young adult; however, in index cases and close contacts of other age groups, transmission did not differ between variants. This may explain the increased proportion of young people who have been infected in the surges due to the Delta variant. The Delta variant was more transmissible than the Alpha variant when the index cases were unvaccinated against COVID-19, and their vaccination equaled the transmissibility of both variants, which suggests a higher impact of vaccination in controlling transmission of the Delta variant.

## INTRODUCTION

Several SARS-CoV-2 variants of concern have circulated since the beginning of the COVID-19 pandemic (1). The Alpha (B.1.1.7) variant has been detected in Europe since late 2020 and has been the main cause of the epidemic surges in the first half of 2021. The Delta (B.1.617.2) variant emerged during 2021, progressively replaced the Alpha variant, and was responsible for most epidemic surges from June to November 2021. In Spain, both variants circulated from June to August 2021 (1, 2).

The rapid spread of the Delta variant in many countries has suggested possible increased transmissibility compared with the Alpha variant (3–6). The Delta variant presents mutations associated with increased infectivity in human cells with angiotensin-converting enzyme 2 receptors (7). Compared with Alpha variant cases, those with Delta variant have shown increased transmissibility (8) and higher risk of hospital admission (9).

Nevertheless, more studies are necessary to clarify pendent aspects. COVID-19 vaccines reduce the risk of Delta variant infection (10), but are less effective against the Delta variant when compared with the Alpha variant (11, 12). The emergence of the Delta variant in Spain was associated with an increased proportion of young people among COVID-19 case (2). Transmission differences between variants are difficult to separate from the effect of other changes, since the Delta variant emerged simultaneously with the increase in vaccination coverage, the subsequent increase in mobility and social interaction, and the relaxation of some preventive measures (13).

The present study aimed to compare the probability of transmission of the Alpha and Delta variants of SARS-CoV-2 from laboratory-confirmed index cases with their close contacts and to investigate characteristics that may modify the susceptibility of the close contacts and the infectiousness of the index cases.

## RESULTS

### Characteristics of close contacts and index cases by variant.

The study included close contacts of index cases with the Alpha variant (*n* = 2,139) and the Delta variant (*n* = 5,439). On average, 3.1 close contacts for each index case (7,578/2,473) were included in this study. Among index cases with the Delta variant, 29.3% were 40 years or older versus 24.1% of those with the Alpha variant, while among their close contacts these percentages were 52.1% and 38.1%, respectively. Index cases with the Delta variant and their close contacts were more frequently vaccinated (37.2% and 61.4%, respectively) than those with the Alpha variant (9.9% and 33.1%, respectively) (Table 1).

**TABLE 1:** Characteristics of the close contacts tested for COVID-19 and their infected index cases

	All close contacts			Unvaccinated close contacts		
	Alpha variant in index case (*n* = 2,139)	Delta variant in index case (*n* = 5,439)		Alpha variant in index case (*n* = 1,430)	Delta variant in index case (*n* = 2,102)	
Characteristics of the index cases and the close contacts	*n* (%)	*n* (%)	*P*	*n* (%)	*n* (%)	*P*
Index case characteristics						
Age groups, yrs			<0.001			<0.001
≤ 5	62 (2.9)	103 (1.9)		54 (3.8)	64 (3.0)	
6–11	66 (3.1)	208 (3.8)		53 (3.7)	117 (5.6)	
12–17	351 (16.4)	992 (18.2)		278 (19.4)	455 (21.6)	
18–39	1,145 (53.5)	2,542 (46.7)		694 (48.5)	1,029 (49.0)	
40–59	438 (20.5)	985 (18.1)		319 (22.3)	335 (15.9)	
≥ 60	77 (3.6)	609 (11.2)		32 (2.2)	102 (4.9)	
COVID-19 vaccination status			<0.001			<0.001
Unvaccinated	1,927 (90.1)	3,414 (62.8)		1,333 (93.2)	1,627 (77.4)	
Vaccinated	212 (9.9)	2,025 (37.2)		97 (6.8)	475 (22.6)	
Close contact characteristics						
Age group, yrs			<0.001			<0.001
≤ 5	144 (6.7)	234 (4.3)		144 (10.1)	234 (11.1)	
6–11	143 (6.7)	349 (6.4)		143 (10.0)	349 (16.6)	
12–17	307 (14.4)	537 (9.9)		305 (21.3)	492 (23.4)	
18–39	730 (34.1)	1,486 (27.3)		649 (45.4)	869 (41.3)	
40–59	550 (25.7)	1,942 (35.7)		179 (12.5)	131 (6.2)	
≥ 60	265 (12.4)	891 (16.4)		10 (0.7)	27 (1.3)	
Sex			0.066			0.020
Male	1,058 (49.5)	2,563 (47.1)		761 (53.2)	1,035 (49.2)	
Female	1,081 (50.5)	2,876 (52.9)		669 (46.8)	1,067 (50.8)	
Major chronic conditions			0.002			0.182
No	1,640 (76.7)	3,980 (73.2)		1,193 (83.4)	1,717 (81.7)	
Yes	499 (23.3)	1,459 (26.8)		237 (16.6)	385 (18.3)	
COVID-19 vaccination			<0.001			
Unvaccinated	1,430 (66.9)	2,102 (38.6)				
Vaccinated	709 (33.1)	3,337 (61.3)				
Contact setting			0.002			0.236
Household	1,082 (50.6)	2,970 (54.6)		633 (44.3)	973 (46.3)	
Non-household	1,057 (49.4)	2,469 (45.4)		797 (55.7)	1,129 (53.7)	
Month of contact			<0.001			<0.001
June	1,649 (77.1)	361 (6.6)		1,178 (82.4)	204 (9.7)	
July	470 (22.0)	2,635 (48.4)		243 (17.0)	1,185 (56.4)	
August	20 (0.9)	2,443 (44.9)		9 (0.6)	713 (33.9)	

### Probabilities of SARS-CoV-2 transmission by variant.

The secondary attack rate was 24% in close contacts of index cases with the Alpha variant and 26% in those exposed to the Delta variant. Among unvaccinated close contacts, the secondary attack rate was higher for the Delta variant (43%) than for the Alpha variant (30%). Secondary attack rates were considerably lower in COVID-19 vaccinated close contacts than in those unvaccinated; however, similar findings were not observed for the vaccination status of index cases. The highest secondary attack rates were observed among unvaccinated close contacts of index cases with the Delta variant when the index case was 40 years or older (50%), the close contact was 18 to 39 years old (49%) and the contact setting was the household (49%) (Tables 2, 3, and Table S1).

**TABLE 2:** Comparison of the risk of transmission between the Delta and Alpha variants of SARS-CoV-2 by characteristics of the close contacts

	All close contacts				Unvaccinated close contacts			
Characteristics of the close contacts and variant	Infections/contacts	SAR %	Adjusted RR (95% CI)^*a*^	*P*	Infections/contacts	SAR %	Adjusted RR (95% CI)^*a*^	*P*
Total								
Alpha variant	506/2,139	24	1		424/1,430	30	1	
Delta variant	1,394/5,439	26	1.32 (1.13 to 1.53)	<0.001	911/2,102	43	1.30 (1.10 to 1.54)	0.002
Aged <18 yrs								
Alpha variant	190/594	32	1		190/592	32	1	
Delta variant	443/1,120	40	0.98 (0.75 to 1.28)	0.879	430/1,075	40	0.98 (0.75 to 1.27)	0.859
Aged 18 to 39 yrs								
Alpha variant	195/730	27	1		182/649	28	1	
Delta variant	521/1,486	35	1.62 (1.29 to 2.03)	<0.001	427/869	49	1.64 (1.30 to 2.08)	<0.001
Aged ≥40 yrs								
Alpha variant	121/815	15	1		52/189	28	1	
Delta variant	430/2,833	15	1.31 (0.97 to 1.79)	0.083	54/158	34	1.26 (0.62 to 2.54)	0.528
Male								
Alpha variant	268/1,058	25	1^*b*^		229/761	30	1	
Delta variant	661/2,563	26	1.21 (0.98 to 1.50)	0.075	451/1,035	44	1.20 (0.95 to 1.52)	0.129
Female								
Alpha variant	238/1,081	22	1		195/669	29	1^*b*^	
Delta variant	733/2,876	26	1.42 (1.15 to 1.75)	0.001	460/1,067	43	1.40 (1.10 to 1.79)	0.007
Major chronic condition								
Alpha variant	108/499	22	1		77/237	33	1	
Delta variant	328/1,459	23	1.17 (0.85 to 1.60)	0.349	155/385	40	1.06 (0.71 to 1.59)	0.763
No major chronic condition								
Alpha variant	398/1,640	24	1		347/1,193	29	1	
Delta variant	1,066/3,980	27	1.37 (1.15 to 1.62)	<0.001	756/1,717	44	1.36 (1.13 to 1.65)	0.001
Household contact								
Alpha variant	346/1,082	32	1		281/633	44	1	
Delta variant	839/2,970	28	1.10 (0.91 to 1.34)	0.324	479/973	49	1.02 (0.80 to 1.29)	0.878
Non-household contact								
Alpha variant	160/1,057	15	1		143/797	18	1	
Delta variant	555/2,469	23	1.71 (1.35 to 2.16)	<0.001	432/1,129	38	1.69 (1.31 to 2.18)	<0.001
Unvaccinated								
Alpha variant	424/1,430	30	1					
Delta variant	911/2,102	43	1.30 (1.10 to 1.54)	0.002				
Vaccinated								
Alpha variant	82/709	12	1					
Delta variant	483/3,337	15	1.34 (0.97 to 1.84)	0.077				

a SAR, secondary attack rate; RR, relative risk; CI confidence interval.

b RR, relative risk adjusted by age group (≤5, 6–11, 12–17, 18–39, 40–59, and  ≥60 years), sex, contact setting (household or other), major chronic conditions, and COVID-19 vaccination status of the close contact, as well as age group, COVID-19 vaccination status, and month of the index case.

**TABLE 3:** Comparison of the risk of transmission between the Delta and Alpha variants of SARS-CoV-2 by age and vaccination status of the infected index cases

	All close contacts				Unvaccinated close contacts			
Characteristics of the index cases and variant	Infections/contacts	SAR %	Adjusted RR (95% CI)^*a*^	*P*	Infections/contacts	SAR %	Adjusted RR (95% CI)^*a*^	*P*
Aged <12 yrs								
Alpha variant	36/128	28	1		35/107	33	1	
Delta variant	63/311	20	0.75 (0.38 to 1.50)	0.419	41/181	23	0.58 (0.28 to 1.23)	0.155
Aged 12 to 39 yrs								
Alpha variant	312/1,496	21	1		263/972	27	1	
Delta variant	908/3,534	26	1.51 (1.27 to 1.79)	<0.001	651/1,484	44	1.46 (1.20 to 1.77)	<0.001
Aged ≥40 yrs								
Alpha variant	158/515	31	1		126/351	36	1	
Delta variant	423/1,594	27	0.80 (0.56 to 1.13)	0.198	219/437	50	0.88 (0.57 to 1.35)	0.551
Unvaccinated^*b*^								
Alpha variant	453/1,927	24	1		392/1,333	29^*b*^	1	
Delta variant	969/3,414	28	1.44 (1.23 to 1.69)	<0.001	718/1,627	44	1.39 (1.16 to 1.66)	<0.001
Vaccinated								
Alpha variant	53/212	25	1		32/97	33	1	
Delta variant	425/2,025	21	0.68 (0.46 to 1.02)	0.682	193/475	41	0.67 (0.40 to 1.12)	0.128

a SAR, secondary attack rate; RR, relative risk; CI confidence interval.

b RR, relative risk adjusted by age group (≤5, 6–11, 12–17, 18–39, 40–59, and ≥60 years), sex, contact setting (household or other), major chronic conditions, and COVID-19 vaccination status of the close contact, as well as age group, COVID-19 vaccination status, and month of the index case.

### Adjusted comparison of the risk of transmission between variants.

**FIG 1:**
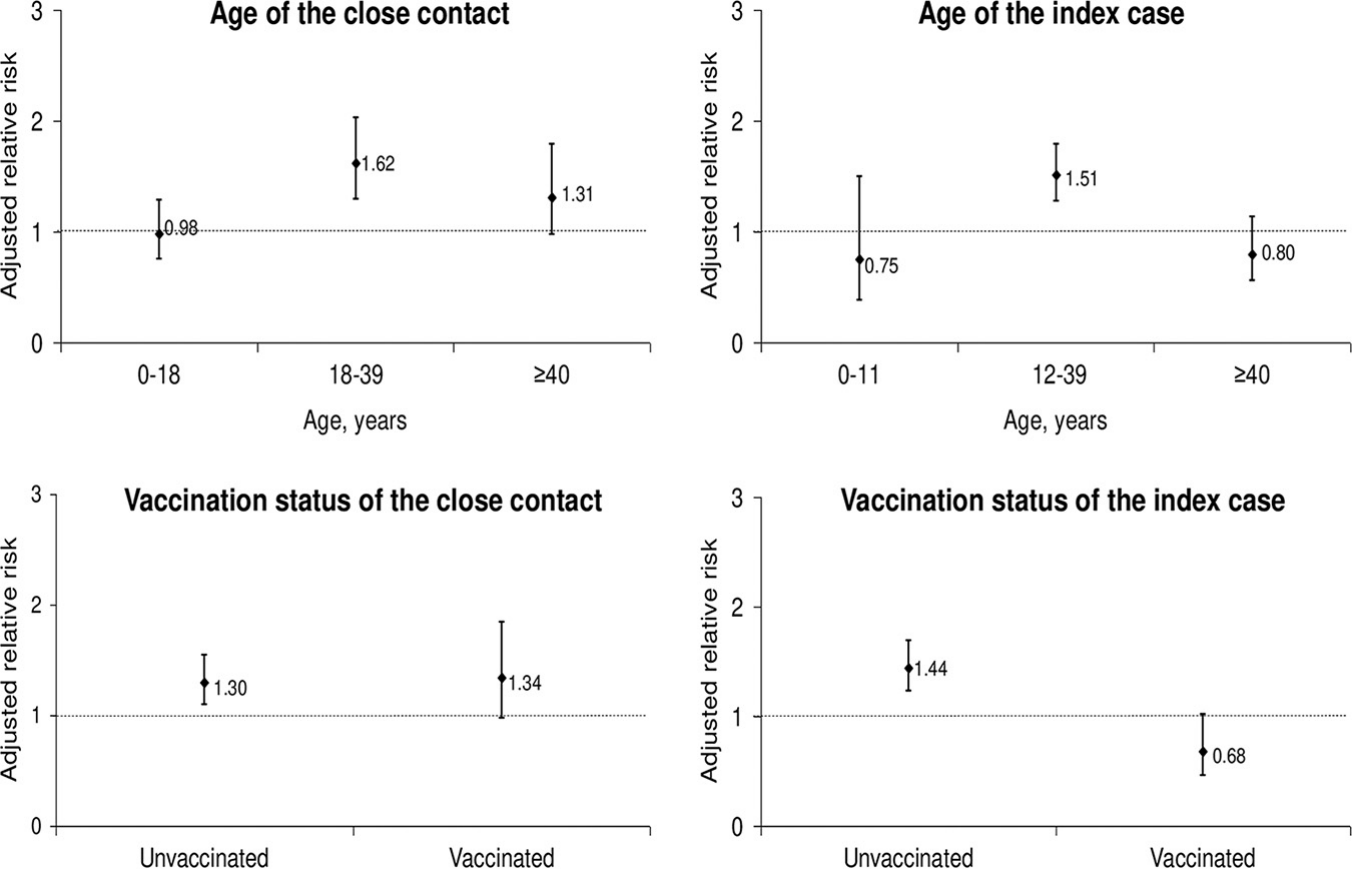
Excess transmission of the SARS-CoV-2 Delta variant compared with the Alpha variant by the age, and the COVID-19 vaccination status of the index case and the close contact. Relative risk adjusted by age, sex, contact setting, major chronic conditions, and COVID-19 vaccination status of the close contact, as well as age group and COVID-19 vaccination status of the index case.

In the overall adjusted analysis, the Delta variant was associated with a 32% higher risk of transmission than the Alpha variant (relative risks [RR] 1.32, 95% confidence interval [CI] = 1.13 to 1.53), and the estimate was similar when vaccinated close contacts were excluded (RR 1.30, 95% CI = 1.10 to 1.54). Excess transmissibility of the Delta variant remained in many of the analyses stratified by relevant covariates. Only the analyses of close contacts of index cases younger than 12 years and aged 40 years or older, of vaccinated index cases, of close household contacts, and close contacts younger than 18 years did not show relevant differences in the transmission between variants (Tables S2 and S3, and Fig. 1).

The Delta variant showed 71% more transmissibility compared to the Alpha variant among non-household close contacts (RR 1.71, 95% CI = 1.35 to 2.16), but this excess transmission was lower (*P_interaction_* < 0.001) and not statistically significant in household contacts (RR 1.10, 95% CI = 0.91 to 1.34).

### Differences in the risk of transmission between variants by age.

Comparisons of transmissibility of the Delta versus the Alpha variant in disaggregated age categories of index cases and close contacts are presented in Table S2 and S3. To simplify the result presentation, some close categories with no significantly different estimates were aggregated in subsequent analyses. The Delta variant was more transmissible than the Alpha variant from index cases aged 12 to 39 years (RR 1.51, 95% CI = 1.27 to 1.79), but this excess transmission disappeared from index cases younger than 12 years (RR 0.75, 95% CI = 0.38 to 1.50; *P_interaction_* = 0.007) or older than 40 years (RR 0.80, 95% CI = 0.56 to 1.13; *P_interaction_* = 0.022) (Table 3). Regarding the age of the close contacts, the excess transmission of the Delta variant was observed in those aged 18 to 39 years (RR 1.62, 95% CI = 1.29 to 2.03), while was not observed in those younger than 18 years (RR 0.98, 95% CI = 0.75 to 1.28; *P_interaction_* = 0.064). Estimates were similar when vaccinated close contacts were excluded from the analysis (RR 1.64, 95% CI = 1.30 to 2.08 and RR 0.98, 95% CI = 0.75 to 1.27, respectively) and interaction reach statistical significance (*P_interaction_* = 0.015). Close contacts older than 40 years also seemed to present an excess transmission of the Delta variant although the estimate was not statistically significant (RR 1.31, 95% CI = 0.97 to 1.79) (Table 2).

### Differences in the risk of transmission between variants by vaccination status.

The Delta variant was more transmissible than the Alpha variant to unvaccinated close contacts (RR 1.30, 95% CI = 1.10 to 1.54) and also to vaccinated close contacts (RR 1.34, 95% CI = 0.97 to 1.84; *P_interaction_* = 0.321). However, the excess transmission of the Delta variant was only present when the index case was unvaccinated (RR 1.44, 95% CI = 1.23 to 1.69), and disappeared when the index case was vaccinated (RR 0.68, 95% CI = 0.46 to 1.02; *P_interaction_* = 0.042) (Tables 2 and 3, and Fig. 1).

### Other analyses of the differences in the risk transmission between variants.

The estimates of the comparisons of transmissibility of both variants did not present statistically significant differences by sex (*P_interaction_* = 0.253), and presence of major chronic conditions (*P_interaction_* = 0.413).

After excluding vaccinated close contacts, the results did not change substantially. The high transmissibility of the Delta variant remained when the index case and the close contact were unvaccinated (RR 1.39, 95% CI = 1.16 to 1.66), which rules out that such a higher transmissibility may be related to the introduction of the COVID-19 vaccination (Table 3).

### COVID-19 vaccination effect in preventing infection by the Alpha and Delta variants.

In close contacts aged 18 years or older, COVID-19 vaccination was similarly effective in preventing infection with the Alpha variant (RR = 0.38, 95% CI = 0.26 to 0.54) and the Delta variant (0.37, 95% CI = 0.31 to 0.44). Vaccination of the index cases did not significantly modify the risk of transmission of the Alpha variant to their close contacts (RR = 1.05, 95% CI = 0.69 to 1.61), but it reduced the risk of transmission of the Delta variant (RR = 0.61, 95% CI = 0.49 to 0.75) (Table 4).

**TABLE 4:** Effect of COVID-19 vaccination of the index case and the close contact in preventing transmission of the Alpha and Delta variants of SARS-CoV-2

Variant in the index case and characteristics of the close contacts	Infections/contacts	Crude RR (95% CI)^*a*^	Adjusted RR (95% CI)^*a*^
Index case infected with the Alpha variant			
Total close contacts ≥18 yrs			
Unvaccinated close contact	234/838	1	1
Vaccinated close contact	82/707	0.42 (0.32 to 0.53)	0.38 (0.26 to 0.54)
Unvaccinated index case	280/1,371	1	1
Vaccinated index case	36/174	1.01 (0.72 to 1.43)	1.05 (0.69 to 1.61)
Close contact aged 18 to 39 yrs			
Unvaccinated close contact	182/649	1	1
Vaccinated close contact	13/81	0.57 (0.33 to 1.01)	0.51 (0.29 to 0.90)
Unvaccinated index case	180/669	1	1
Vaccinated index case	15/61	0.91 (0.54 to 1.55)	0.96 (0.53 to 1.75)
Close contact aged ≥40 yrs			
Unvaccinated close contact^*b*^	52/189	1	1
Vaccinated close contact	69/626	0.40 (0.28 to 0.57)	0.40 (0.26 to 0.62)
Unvaccinated index case	100/702	1	1
Vaccinated index case	21/113	1.31 (0.82 to 2.09)	1.18 (0.64 to 2.16)
Index case infected with the Delta variant			
Total close contacts ≥18 yrs			
Unvaccinated close contact	481/1,027	1	1
Vaccinated close contact	470/3,292	0.31 (0.27 to 0.35)	0.37 (0.31 to 0.44)
Unvaccinated index case	681/2,644	1	1
Vaccinated index case	270/1,675	0.63 (0.54 to 0.72)	0.61 (0.49 to 0.75)
Close contact aged 18 to 39 yrs			
Unvaccinated close contact	427/869	1	1
Vaccinated close contact	94/617	0.31 (0.25 to 0.39)	0.39 (0.30 to 0.51)
Unvaccinated index case	450/1,053	1	1
Vaccinated index case	71/433	0.38 (0.30 to 0.49)	0.47 (0.34 to 0.65)
Close contact aged ≥40 yrs			
Unvaccinated close contact	54/158^*b*^	1	1
Vaccinated close contact	376/2,675	0.41 (0.31 to 0.55)	0.47 (0.35 to 0.64)
Unvaccinated index case	231/1,591	1	1
Vaccinated index case	199/1,242	1.10 (0.91 to 1.33)	0.74 (0.55 to 1.00)

a RR, relative risk; CI, confidence interval.

b RR, relative risk adjusted by age group (≤5, 6–11, 12–17, 18–39, 40–59, and ≥60 years), sex, contact setting (household or other), and major chronic conditions of the close contact, and age group, COVID-19 vaccination status, and month of the index case.

## DISCUSSION

The present study confirms that, on average, the Delta variant is more transmissible than the Alpha variant as several authors had described (9, 14). Although the excess transmission of the Delta variant was moderate (32%), this advantage may be sufficient to explain the replacement of the circulation of the Alpha variant by the Delta variant, as it has happened in many countries in the course of the pandemic (8, 15).

We observed that the excess transmission of the Delta variant in comparison with the Alpha variant was more pronounced in non-household contacts (71%) and almost disappeared in household contacts (10%). This can be explained because among household contacts the exposure is usually more intense and repeated, leading to an equally high risk of infection, although the risk associated with a single exposure was lower; however, among non-household contacts, differences in the transmissibility of the variants could lead to different results.

According to our results, the Delta variant showed increased infectivity when the index case was an adolescent or young adult (12 to 39 years old); however, in other age groups there was no difference in infectivity between cases with Alpha and Delta variants. Furthermore, young adults (18 to 39 years old) were more susceptible to infection from index cases with the Delta variant than with the Alpha variant, while this difference was smaller and not statistically significant in close contacts of other age groups. At the beginning of the pandemic, SARS-CoV-2 infection showed a lower preference for transmission among adolescents and young adults (16), and this pattern continued throughout 2020 (17). In summer 2021, coinciding with the introduction of the Delta variant in Spain, the incidence of COVID-19 increased markedly in young people (18). Our findings also suggest that the Delta variant could have contributed to increased transmissibility among adolescents and young adults, while maintaining similar transmissibility among people of other ages (19).

The Delta variant was more transmissible than the Alpha variant when the index case or close contacts were not vaccinated against SARS-CoV-2. Several studies have suggested slightly lower COVID-19 vaccine effectiveness in preventing cases caused by the Delta variant compared with those caused by the Alpha variant (12, 13, 20), but in the present study, we do not detect significant differences. Furthermore, we found that in vaccinated people the differences in susceptibility between these variants were maintained and the differences in susceptibility were reduced, which means that the impact of vaccination on the control of SARS-CoV-2 may be similar or even greater against the Delta variant than against the Alpha variant. People infected with the Delta variant have been found to have greater viral shedding compared with people infected with the Alpha variant (6, 21, 22). Given that vaccination against COVID-19 reduces viral shedding (23), it would be interesting to study this reduction in people infected with the Delta variant. Our results suggest that the progressive vaccination of all age groups will tend to reduce the differences in transmissibility between these variants.

The strengths of this study are that it compared the transmission of the Delta and Alpha variants in a cohort of close contacts studied with the same protocol for months with circulation of both variants. All participants had a similar exposure with a high risk of infection as they were close contacts of an infected index case. This study provides good representativeness of the general population. In addition, two different contact situations have been included (household and non-household contact), which provide two complementary perspectives of SARS-CoV-2 transmission in the population. We obtained the COVID-19 vaccination status from the regional vaccination registry and other variables from the electronic medical records and the enhanced epidemiological surveillance of COVID-19. The study was limited to the population with stable residence in the region to avoid bias due to incomplete information. The study period included only the 3-month period with co-circulation of Alpha and Delta variants, and analyses were adjusted for month and age to control the confounding effect due to changes in non-pharmaceutical interventions or in compliance with preventive measures by the population.

This study has some limitations. Results of the quantitative reverse transcription-PCR (RT-qPCR) TaqPath and TaqMan assays are proxies of SARS-CoV-2 variants, whose definitive classification should be based on whole genome sequencing; therefore, misclassification of variants may be possible. As the SARS-CoV-2 variant was only assessed in index cases with low cycle-threshold value, cases with the lowest transmissibility may be less represented. Symptomatic close contacts with a positive antigen test were also considered as infected because the specificity of this test has been shown to be high in these patients (24). This study was conducted under specific epidemiological and vaccination conditions and results may vary at other sites. Because people with previous COVID-19 were excluded, reinfections are not represented in the results.

In conclusion, the Delta variant showed higher transmissibility than the Alpha variant when the index case was an adolescent or young adult and when the close contact was a young adult; however, in index cases and close contacts of other age groups the transmissibility did not differ. This may explain the increase in the proportion of young people who have been infected in the surges due to the Delta variant. The Delta variant was more transmissible than the Alpha variant when the index case was unvaccinated for COVID-19; nevertheless, vaccination of the index case equalized the transmissibility of both variants, suggesting a greater impact of vaccination in reducing transmission of the Delta variant. These results introduce interesting hypothesis of the host-agent interaction to be studied.

## MATERIALS AND METHODS

### Ethics statement.

This study was approved by the Ethical Committee for Clinical Research of Navarre, which waived the requirement of obtaining informed consent (approval code: PI2020/45).

### Design, setting, and data source.

This prospective cohort study was based on the activities of contact tracing of COVID-19 cases from June 2021 to August 2021 in Navarra, Spain.

As part of the pandemic control measures, all confirmed COVID-19 cases were interviewed to identify their close contacts (20, 25, 26). The index case was the first person who presented COVID-19 and was confirmed by RT-qPCR or antigen test in a specific setting. Close contact was defined as any person who had a high-risk exposure to a confirmed COVID-19 index case in a period ranging from 48 h before the onset of symptoms of the case, to 10 days after the onset of symptoms, or in the 2 days before the sampling leading to confirmation, to 10 days after sampling for asymptomatic cases (26). A high-risk exposure was considered to have spent more than 15 min without a face mask at a distance lower than 1.5 m. Close contacts were preferably tested twice, immediately and on day 10 after last exposure to risk, and at least once after day 7 using a commercial RT-qPCR tests for SARS-CoV-2, usually Allplex 2019-nCoV assay (Seegene, South Korea), in nasopharyngeal samples. In symptomatic close contacts, a positive result of a commercial antigen test performed by a health care professional within 5 days from the symptom onset was also considered confirmatory, but close contacts with negative antigen test were retested with RT-qPCR (27). Contact tracing was documented in a register that included information of the index case and the close contact, and was electronically connected through the individual identification number with the databases of test results, electronic medical records and enhanced epidemiological surveillance of COVID-19.

Index case samples with cycle threshold value ≤30 were tested by TaqPath COVID-19 RT-PCR kit and TaqMan SARS-CoV-2 Mutation Panel (Thermo Fisher Scientific, USA) to get an approximation of the variant. Because the Alpha and Delta variants predominated during the study period, the variant analysis was limited to the identification of both variants. Spike gene target failure by TaqPath was used as a proxy measure of the Alpha variant (28). The detection of the L452R mutation by TaqMan assay was used as a proxy measure of the Delta variant.

COVID-19 vaccination campaign included BNT162b2 mRNA (BioNTech-Pfizer), mRNA-1273 (Moderna), ChAdOx1 nCoV-19 (Oxford-AstraZeneca), and Ad26.COV2-S vaccines (Janssen). The vaccination status of index cases and their close contacts were obtained from the regional vaccination register. Vaccination was considered 14 days after administration (29).

### Study population.

The present analysis included close contacts of COVID-19 index cases confirmed between June 2021 and August 2021 and classified as infected by the Alpha or Delta variants. Close contacts without residence in the region, with a previous SARS-CoV-2 infection, nursing home residents, and those who did not complete the testing protocol were excluded. Household close contacts were considered those who lived in the same home for at least one night during the infectivity period of the index case.

### Statistical analysis.

The incidence of SARS-CoV-2 infection in the close contacts (secondary attack rate) was compared according to the variant detected in the index case, and also was stratified by the other covariables.

The risk of transmission of the Delta variant compared with that of the Alpha variant was assessed by multivariate Cox regression models. The same risk period was assigned to everyone in the cohort; therefore, the Cox regression provided estimates of the adjusted RR with 95% CI. Adjusted models included the age group (≤5, 6–11, 12–17, 18–39, 40–59, and ≥60 years), sex, presence of major chronic conditions, COVID-19 vaccination status, and contact setting (household or non-household) of the close contacts, as well as the age group, month of diagnosis and COVID-19 vaccination status of the index case. The interaction terms between each covariable and the variant were tested. The adjusted comparison of variants was repeated for each category of the mentioned covariables. The analyses were repeated including only unvaccinated close contacts to rule out the possible interference of the vaccination status on the results. Among close contacts aged 18 years or older, the vaccination effect in preventing SARS-CoV-2 infection was evaluated according to the variant identified in the index case. Similarly, among index cases aged 18 years or older, the vaccination effect in preventing SARS-CoV-2 transmission was evaluated by variant.
