# Supplementary material for: Differences in Transmission between SARS-CoV-2 Alpha (B.1.1.7) and Delta (B.1.617.2) Variants
Source: Microbiol Spectr. 2022 Apr 12;10(2):e00008-22. doi: 10.1128/spectrum.00008-22 (PMC9045255; doi:10.1128/spectrum.00008-22)
Supplement: SUPPLEMENTAL FILE 1 — Supplemental material. Download spectrum.00008-22-s0001.pdf, PDF file, 0.3 MB [file spectrum.00008-22-s0001.pdf]

SUPPLEMENTARY MATERIAL

Trobajo Sanmartín C, et al. Differences in transmission between SARS-CoV-2 Alpha (B.1.1.7) and Delta (B.1.617.2) variants. *Microbiology Spectrum*.

Table S1. Secondary attack rates by SARS-CoV-2 variant, and vaccination status of the index case, and age and vaccination status of the close contact.

Close contact vaccination status and age	All close contacts		Close contacts of vaccinated index case		Close contacts of unvaccinated index case	
	Infections / contacts	SAR %	Infections / contacts	SAR %	Infections / contacts	SAR %
**Unvaccinated close contact**						
**Total**						
Alpha variant	424/1430	30	32/97	33	392/1333	29
Delta variant	911/2102	43	193/475	41	718/1627	44
**Aged <18 years**						
Alpha variant	190/592	32	17/38	45	173/554	31
Delta variant	430/1075	40	146/325	45	284/750	38
**Aged 18 to 39 years**						
Alpha variant	182/649	28	12/50	24	170/599	28
Delta variant	427/869	49	37/111	33	390/758	52
**Aged ≥40 years**						
Alpha variant	52/189	28	3/9	33	49/180	27
Delta variant	54/158	34	10/39	26	44/119	37
**Vaccinated close contact**						
**Total**						
Alpha variant	82/709	12	21/115	18	61/594	10
Delta variant	483/3337	15	232/1550	15	251/1787	14
**Aged <18 years**						
Alpha variant	0/2	0	0/0	NA	0/2	0
Delta variant	13/45	29	9/25	36	4/20	20
**Aged 18 to 39 years**						
Alpha variant	13/81	16	3/11	27	10/70	14
Delta variant	94/617	15	34/322	11	60/295	20
**Aged ≥40 years**						
Alpha variant	69/626	11	18/104	17	51/522	10
Delta variant	376/2675	14	189/1203	16	187/1472	13

SAR, secondary attack rate; NA, not available.

Table S2. Comparison of the risk of transmission between Delta and Alpha (reference) variants of SARS-CoV-2 by age of the close contacts.

	All close contacts				Unvaccinated close contacts			
**Age of the close contact and variant**	**Infections / contacts**	**SAR %**	**Adjusted RR (95% CI)** [a]	***P***	**Infections / contacts**	**SAR %**	**Adjusted RR (95% CI)** [a]	***P***
**0 to 5 years**								
Alpha variant	43/144	30	1		43/144	30	1	
Delta variant	84/234	36	0.64 (0.33-1.25)	0.191	84/234	36	0.64 (0.33-1.25)	0.191
**6 to 11 years**								
Alpha variant	55/143	39	1		55/143	39	1	
Delta variant	146/349	42	1.26 (0.73-2.16)	0.404	146/349	42	1.26 (0.73-2.16)	0.404
**12 to 17 years**								
Alpha variant	92/307	30	1		92/305	30	1	
Delta variant	213/537	40	1.09 (0.76-1.55)	0.652	200/492	41	1.08 (0.75-1.54)	0.691
**18 to 39 years**								
Alpha variant	195/730	27	1		182/649	28	1	
Delta variant	521/1486	35	1.62 (1.29-2.03)	<0.001	427/869	49	1.64 (1.30-2.08)	<0.001
**40 to 59 years**								
Alpha variant	84/550	15	1		48/179	27	1	
Delta variant	274/1942	14	1.29 (0.89-1.87)	0.176	46/131	35	1.19 (0.55-2.56)	0.663
**≥60 years**								
Alpha variant	37/265	14	1		4/10	40	1	
Delta variant	156/891	18	1.35 (0.78-2.38)	0.286	8/27	30	0.81 (0.05-13.34)	0.883

SAR, secondary attack rate; RR, relative risk; CI confidence interval.

[a] RR, relative risk adjusted by age group (≤5, 6–11, 12–17, 18–39, 40–59 and ≥60 years), sex, contact setting (household or other), major chronic conditions and COVID-19 vaccination status of the close contact, and age group, COVID-19 vaccination status and month of the index case.

Table S3. Comparison of the risk of transmission between Delta and Alpha (reference) variants of SARS-CoV-2 by age of the infected index cases.

	All close contacts				Unvaccinated close contacts			
**Index case age and variant**	**Infections / contacts**	**SAR %**	**Adjusted RR (95% CI) [a]**	***P***	**Infections / contacts**	**SAR %**	**Adjusted RR (95% CI) [a]**	***P***
**0 to 5 years**								
Alpha variant	16/62	26	1		15/54	28	1	
Delta variant	21/103	20	0.17 (0.02-2.03)	0.162	13/64	20	0.24 (0.02-2.97)	0.265
**6 to 11 years**								
Alpha variant	20/66	30	1		20/53	38	1	
Delta variant	42/208	20	0.80 (0.39-1.65)	0.547	28/117	24	0.61 (0.28-1.33)	0.214
**12 to 17 years**								
Alpha variant	60/351	17	1		56/278	20	1	
Delta variant	239/992	24	1.64 (1.09-2.47)	0.017	179/455	39	1.64 (1.06-2.53)	0.026
**18 to 39 years**								
Alpha variant	252/1145	22	1		207/694	30	1	
Delta variant	669/2542	26	1.48 (1.22-1.79)	<0.001	472/1029	46	1.42 (1.14-1.76)	0.002
**40 to 59 years**								
Alpha variant	134/438	31	1		115/319	36	1	
Delta variant	270/985	27	0.85 (0.56-1.28)	0.424	174/335	52	0.86 (0.53-1.37)	0.513
**≥60 years**								
Alpha variant	24/77	31	1		11/32	34	1	
Delta variant	153/609	25	0.80 (0.41-1.55)	0.509	45/102	44	1.28 (0.44-3.76)	0.652

SAR, secondary attack rate; RR, relative risk; CI confidence interval.

[a] RR, relative risk adjusted by age group (≤5, 6–11, 12–17, 18–39, 40–59 and ≥60 years), sex, contact setting (household or other), major chronic conditions and COVID-19 vaccination status of the close contact, and age group, COVID-19 vaccination status and month of the index case.
